# Supplementary material for: Polycaprolactone (PCL)-Polylactic Acid (PLA)-Glycerol (Gly) Composites Incorporated with Zinc Oxide Nanoparticles (ZnO-NPs) and Tea Tree Essential Oil (TTEO) for Tissue Engineering Applications
Source: Pharmaceutics. 2022 Dec 22;15(1):43. doi: 10.3390/pharmaceutics15010043 (PMC9864333; doi:10.3390/pharmaceutics15010043)
Supplement: Supplementary file 1 [file pharmaceutics-15-00043-s001.zip › pharmaceutics-2101812-supplementary.pdf]

pharmaceutics

MDPI

Article

Polycaprolactone (PCL)-Polylactic Acid (PLA)-Glycerol (Gly) Composites Incorporated with Zinc Oxide nanoparticles (ZnO-NPs) and Tea Tree Essential Oil (TTEO) for Tissue Engineering Applications

Carlos David Grande-Tovar [1,*], Jorge Iván Castro [2], Carlos Humberto Valencia Llano [3], Diego López Tenorio [3], Marcela Saavedra [4], Paula A. Zapata [4] and Manuel N. Chaur [2,*]

1 Grupo de Investigación de Fotoquímica y Fotobiología, Universidad del Atlántico, Carrera 30 Número 8-49, Puerto Colombia 081008, Colombia
2 Laboratorio SIMERQO, Departamento de Química, Universidad del Valle, Calle 13 No. 100-00, Cali 76001, Colombia
3 Grupo Biomateriales Dentales, Escuela de Odontología, Universidad del Valle, Calle 4B # 36-00, Cali 76001, Colombia
4 Grupo de Polímeros, Facultad de Química y Biología, Universidad de Santiago de Chile, USACH, Santiago 9170020, Chile
* Correspondence: carlosgrande@mail.uniatlantico.edu.co (C.D.G.-T.); manuel.chaur@correounivalle.edu.co (M.N.C.); Tel.: +57-5-3599-484 (C.D.G.-T.)

Citation: Grande-Tovar, C.D.; Castro, J.I.; Valencia Llano, C.H.; Tenorio, D.L.; Saavedra, M.; Zapata, P.A.; Chaur, M.N. Polycaprolactone (PCL)-Polylactic Acid (PLA)-Glycerol (Gly) Composites Incorporated with Zinc Oxide nanoparticles (ZnO-NPs) and Tea Tree Essential Oil (TTEO) for Tissue Engineering Applications. *Pharmaceutics* **2022**, *14*, x. https://doi.org/10.3390/xxxxx

Academic Editor: Helena P. Felgueiras

Received: 30 November 2022
Revised: 15 December 2022
Accepted: 19 December 2022
Published: date

Copyright: © 2022 by the authors. Submitted for possible open access publication under the terms and conditions of the Creative Commons Attribution (CC BY) license (https://creativecommons.org/licenses/by/4.0/).

Abstract: The search for new biocompatible materials that can replace invasive materials in biomedical applications has increased due to the great demand derived from accidents and diseases such as cancer in various tissues. In this sense, four formulations based on polycaprolactone (PCL) and polylactic acid (PLA) incorporated with zinc oxide nanoparticles (ZnO-NPs) and tea tree essential oil (TTEO) were prepared. The sol-gel method was used for zinc oxide nanoparticle synthesis with an average size of 11 ± 2 nm and spherical morphology. On the other hand, Fourier Transformed infrared spectroscopy (FTIR) showed characteristic functional groups for each composite component. The TTEO incorporation in the formulations was related to the increased intensity of the C-O-C band. The thermal properties of the materials show that the degradative properties of the ZnO-NPs decrease the thermal stability. The morphological study by scanning electron microscopy (SEM) showed that the presence of TTEO and ZnO-NPs act synergistically, obtaining smooth surfaces, whereas membranes with the presence of ZnO-NPs or TTEO only show porous morphologies. Histological implantation of the membranes showed biocompatibility and biodegradability after 60 days of implantation. This degradation occurs through the fragmentation of the larger particles with the presence of connective tissue constituted by type III collagen fibers, blood vessels, and inflammatory cells, where the process of resorption of the implanted material continues.

Keywords: biocompatibility; zinc oxide nanoparticles; tissue engineering; nanocomposites

According to our GC-MS analysis, TTEO has 52 compounds previously identified and reported [1].

Table S1. Volatile compounds for the oil essential *Melaleuca alternifolia are* expressed as percentage area.

	Compound	RT	Relative Amount (%)
**Monoterpenes hydrocarbons**	α-Thujene	15.96	1.1
	α-Pinene	16.35	2.7
	Sabinene	18.06	0.1
	β-Pinene	18.33	0.8
	β-Myrcene	18.72	0.8
	α-Phellandrene	19.54	0.5
	α-Terpinene	20.08	9.4
	p-Cymene	20.39	4.7
	Limonene	20.57	1.0
	1,8-cineole	20.75	3.9
	γ-Terpinene	21.91	18.4
**Monoterpenes oxygenated**	Terpinolene	22.93	3.7
	p-Cymenene	23.09	0.1
	Linalool	23.37	0.1
	cis-p-Ment-2-en-1-ol	24.50	0.2
	trans-p-Ment-2-en-1-ol	25.34	0.1
	Terpinene-4-ol	27.09	35.5
	α-Terpineol	27.41	3.5
	Trans-piperitol	27.80	0.1
**Sesquiterpenes oxygenated**	A mixture of compounds $C_{10}H_{16}O$+$C_{10}H_{18}O$+N.I.	29.53	0.1
	A mixture of compounds *trans*-ascaridol glicol+$C_{10}H_{14}O$	30.19	0.1
	N.I.	30.80	0.1
	$C_{10}H_{18}O_2$	31.83	0.1
**Sesquiterpenes no oxygenated**	α–cubebene	32.81	0.1
	Isoledene	33.82	0.1
	α–copaene	34.02	0.2
	α–gurjunene	35.26	0.6
	trans-β-Caryophellene	35.75	0.6
	$C_{15}H_{24}$	36.00	0.1
	α–Maaliene	36.29	0.1
	Aromadendrene	36.44	1.8
	Seline-5,11-diene	36.65	0.2
	Trans-muurola-3,5-diene	36.74	0.2
	α-humulene	37.01	0.2
	Aloaramadendrene	37.18	0.7
	Trans-cadine-1(6),4-diene	37.47	0.4
	γ-muurolene	37.54	0.1
	δ-selinene	37.98	0.3
	Viridiflorene	38.17	1.5
	Bicyclegermacrene	38.32	0.7
	δ-cadidine	38.95	1.6
	Mixture of compounds: *cis*-calamenene+zonarene	39.13	0.7
	Trans-cadine-1,4-diene	39.42	0.3
	epi-globulol	40.42	0.1
	A mixture of compounds: Maaliol-Palustrol	40.68	0.1
	Espatulenol	40.84	0.2
	Globulol	41.14	0.5
	Mixture of compounds: Viridiflorol+cubeban-11-ol	41.41	0.4
	Rosifoliol	41.70	0.2
	$C_{15}H_{24}0$	42.18	0.2
	1-*epi*-cubenol	42.25	0.3
	Cis-cadin-4-en-7-ol	42.66	0.7
